# Effect of Shakuyaku‐kanzo‐to in patients with muscle cramps: A systematic literature review

**DOI:** 10.1002/jgf2.302

**Published:** 2020-02-16

**Authors:** Koshi Ota, Keisuke Fukui, Eriko Nakamura, Masahiro Oka, Kanna Ota, Masahide Sakaue, Yohei Sano, Akira Takasu

**Affiliations:** ^1^ Department of Emergency Medicine Osaka Medical College Takatsuki Japan; ^2^ Research and Development Center Osaka Medical College Takatsuki Japan

**Keywords:** cirrhosis, lumbar spinal stenosis, muscle cramps, Shakuyaku‐kanzo‐to, systematic review

## Abstract

**Background:**

Previous clinical studies have reported that Shakuyaku‐kanzo‐to (SKT) has a therapeutic effect on muscle cramps, but few studies have clarified how SKT acts to treat muscle cramps. The aim of this study was to perform an updated systematic review of clinical trials for SKT in patients with muscle cramps.

**Methods:**

The literature was systematically reviewed to assess the effects of SKT in patients with muscle cramps. PubMed, Web of Science, Cochrane Library, Google Scholar, and Ichushi‐Web were searched using the terms “Shakuyaku‐kanzo‐to” (“shakuyakukanzoto”, etc), “clinical trials” and “muscle cramps”. Two quality assessments were conducted independently by three authors. Data were extracted using a standardized extraction tool, and a qualitative synthesis of evidence was performed.

**Results:**

Three randomized controlled articles were identified and enrolled in this study. A systematic review, but not a meta‐analysis, was performed because of the high heterogeneity and limited number of studies. In patients with liver cirrhosis, the odds ratio (OR) for improvement with SKT compared to placebo was 1.27 (95% confidence interval [CI], 0.445‐2.086) and compared to Goshajinkigan was 0.81 (95%CI, −1.734‐0.114). The OR for improvement with SKT compared with eperisone hydrochloride in patients with lumbar spinal stenosis was 2.86 (95%CI, 0.980‐4.744).

**Conclusions:**

Current evidence appears insufficient to allow a meta‐analysis of the effects of SKT, but SKT might show efficacy in treating muscle cramps in patients with cirrhosis or lumbar spinal stenosis.

## Graphical abstract

The present systematic review included only three clinical trials that investigated the efficacy of Shakuyaku‐kanzo‐to against muscle cramps. Shakuyaku‐kanzo‐to showed efficacy in treating muscle cramps in patients with cirrhosis or lumbar spinal stenosis.

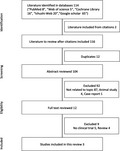

## 1 BACKGROUND

Muscle cramps are painful, spasmodic, involuntary, hard contraction of skeletal muscles that typically occur during or immediately after exercise, usually affecting muscles of the calf or foot. Muscle cramps are considered to represent an alteration of muscle relaxation. About 50%‐60% of healthy adults experience muscle cramps, and the incidence increases with aging and during exercise.^1^ Muscle cramps can be idiopathic or secondary to other medical conditions, with the former being the most common. Secondary causes include structural disorders or leg positioning; neurologic disorders; metabolic disorders, including extracellular fluid volume depletion and electrolyte disturbances; and medications. The latest scientific research suggests the primary cause of muscle cramps involves spinal pathways rather than peripheral excitation of the motor neurons, although the etiology remains unclear.^1^, ^2^

The Japanese traditional herbal medicine Shakuyaku‐kanzo‐to (SKT) represents an equal combination of the roots of *Radix paeoniae* (peony) and *R glycyrrhizae* (licorice), and has long been used for the treatment of muscle cramps in Kampo medicine (Japanese traditional medicine). Two components of SKT can promote an efflux of potassium ions and inhibit the intracellular influx of calcium ions by inhibiting Ca^2+^‐activated K^+^ channels.^3^, ^4^, ^5^ Although the mechanisms underlying the actions of SKT in the inhibition of muscular contraction remain unclear, SKT can act on receptors at neuromuscular synapses with an antispasmodic effect and may act on spinal pathways with antinociceptive effects. While several animal studies and case reports have investigated the effectiveness of SKT, clinical evidence is needed to clarify how SKT is effective against muscle cramps. We therefore aimed to prepare an updated systematic review of SKT in the treatment of muscle cramps.

## 2 METHODS

### 2.1 Search strategy

A search strategy was developed using PubMed, Web of Science, Cochrane Library, Ichushi‐Web, and Google Scholar without language limitations in January 2019. Searches were made with filtering for the following keywords: “Shakuyaku‐kanzo‐to”, “Shakuyakukanzoto”, “Shakuyakukanzo‐to”, “Shakuyakukanzo to”, “Shakuyaku kanzo to”; “muscle cramps”; and “Clinical trial”. AND and NOT “animal study” were applied to a database to create subsets of search results. Citations of studies obtained in the search were also comprehensively reviewed.

### 2.2 Study selection

#### 2.2.1 Inclusion criteria

All studies investigating the efficacy of SKT in the treatment of muscle cramps were included, because the number of studies into SKT use was small.

#### 2.2.2 Exclusion criteria

Studies meeting any of the following criteria were excluded: (a) studies for which the full text was not available in English or Japanese; (b) studies focusing on topics other than use of SKT; (c) studies focusing on topics other than muscle cramps; (d) animal studies; and (e) reviews, letters, or editorials.

### 2.3 Data extraction and quality assessment

The PRISMA guideline and Cochrane Handbook for Systematic Reviews of Interventions were used when searching articles.^6^ We published the protocol for this systematic review in the PROSPERO database (identifier: CRD 42019123160). The following data were extracted from eligible studies: (a) study characteristics (authors, year of publication, institution and country of the study, study period, number of patients, and study design [randomized vs nonrandomized]); (b) demographic characteristics (patient age and enrolled population); and (c) dose of SKT. Eligible articles were fully screened by four reviewers (K.O, E.N, M.O, and Y.S). All disagreements were solved as consensus decisions following discussion. The methodological quality of the selected studies was assessed using Cochrane risk‐of‐bias criteria (Cochrane Collaboration; http://www.cochrane.org/) and a modified version of the assessment checklist developed by Downs and Black.^7^

### 2.4 Data synthesis and statistical analysis

The primary outcome examined in this study was the efficacy of SKT against muscle cramps. The incidence of adverse events associated with SKT was evaluated as an additional outcome. Calculation of these outcomes was attempted using a random‐effects regression model in accordance with the methods of DerSimonian and Laird, revealing only three studies with different background considered unsuitable for network meta‐analysis. Odds ratios (ORs) of clinical results (improvement rate) were examined for the SKT group compared with a placebo group, Goshajinkigan (GJG) group, or eperisone hydrochloride group, but characteristics of patients differed.

Heterogeneity was not tested for, because the number of included studies was small and meta‐analysis could not be performed. The presence of publication bias was likewise not evaluated, because only three studies were included in the systematic review. The need for ethics approval was waived for this systematic review, because only indirect literature was included and evaluated.

## 3 RESULTS

### 3.1 Study characteristics

**Figure 1:**
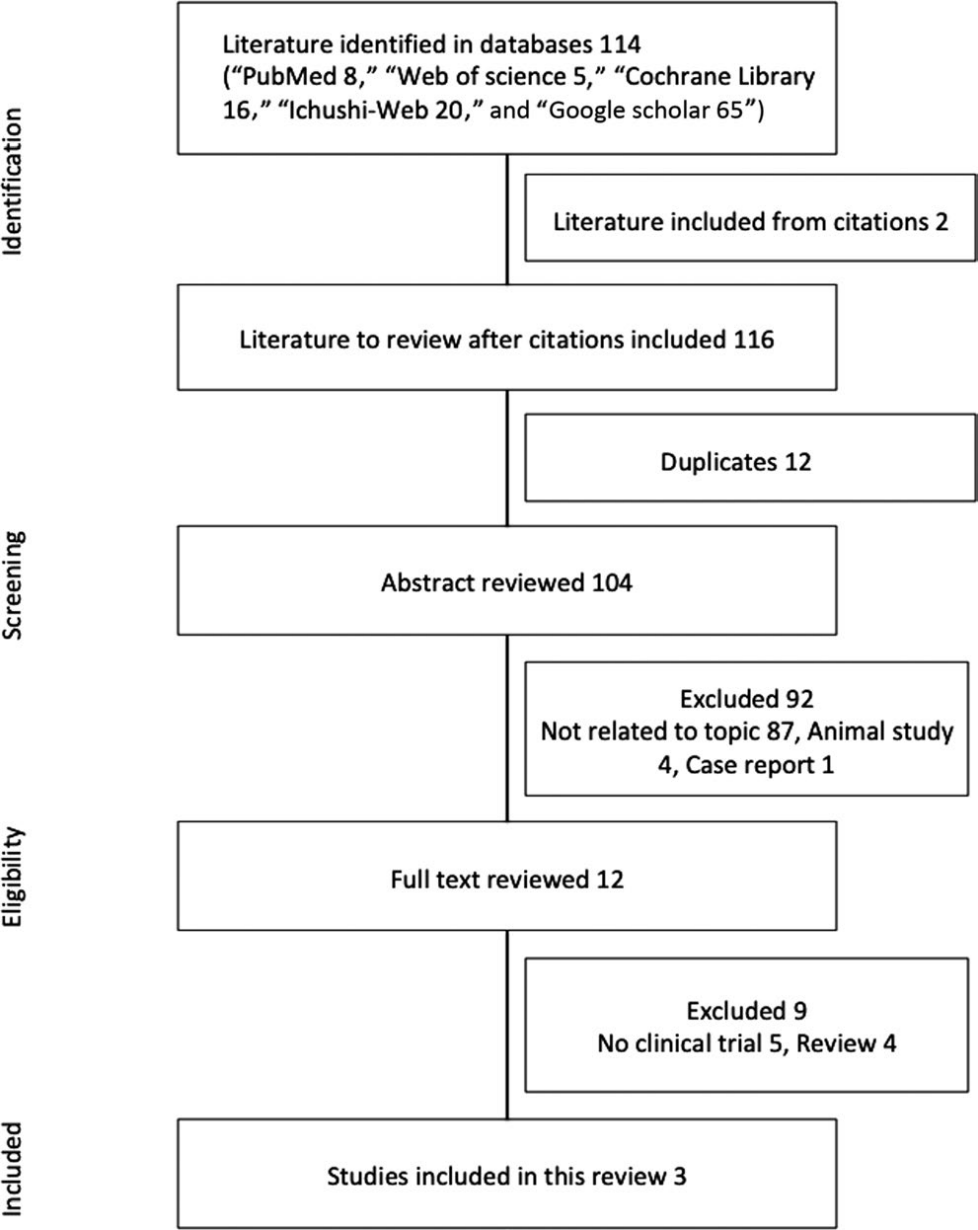
Study flow diagram. Flow diagram of the study selection process and specific reasons for exclusion from the systematic review

Searches identified a total of 116 records, with the following breakdown: PubMed, eight articles; Web of Science, five articles; Cochrane Library, 16 articles; Ichushi‐Web, 20 articles; and Google Scholar, 65 articles. Two other articles were identified from references provided in those selected studies. After removing duplicate records, 72 articles remained. These articles were screened by title and abstract against the inclusion and exclusion criteria, leading to the final inclusion of three articles (Figure 1).

All three studies were conducted in Japan. Blinded and nonblinded randomized controlled trials of SKT in patients with muscle cramps were included.

Age distributions were similar in the three studies, but patient background characteristics differed (Table 1). Patients with cirrhosis were the target population in the studies of Kumada et al^8^ and Nishizawa et al,^9^ whereas patients with lumbar spinal stenosis were the target in the study by Takao et al.^10^

**Table 1:** Characteristics of identified clinical trials for Shakuyaku‐kanzo‐to

Author (y)	Study design	Total n	Patients	Duration (wk)	Age (y) (mean ± SD)	Male (%)	Drug, dose	n	Outcomes	Adverse events
Kumada et al^8^	Randomized double‐blind placebo‐controlled parallel study	101	Cirrhosis	2	59.9 ± 8.4	40.4	SKT 7.5 g/d	52	67.30%	14.30%
				2	60.3 ± 8.3	53.1	Placebo	49	36.70%	4.90%
Nishizawa et al^9^	Randomized controlled trial	75	Cirrhosis	12	62.7 ± 9.5	83.8	SKT 50 mg/kg/d	37	40.50%	16.22%
				12	64.8 ± 10.3	81.6	GJG 90 mg/kg/d	38	60.50%	0%
Takao et al (2015)^10^	Randomized not blinded clinical trial	30	Lumbar spinal stenosis	2	67.9 ± 8.6	56.3	SKT 7.5 g/d	16	87.50%	6.25%
				2	66.7 ± 9.5	50	Eperisone	14	28.57%	0%

Note

Treatment data for study participants. Summary of literature included in the systematic review.

Abbreviations: GJG, Goshajinkigan; SKT, Shakuyaku‐kanzo‐to.

Meta‐analysis was not feasible because of the high heterogeneities and limited number of studies, so only a systematic review was performed.

### 3.2 Outcomes

**Figure 2:**
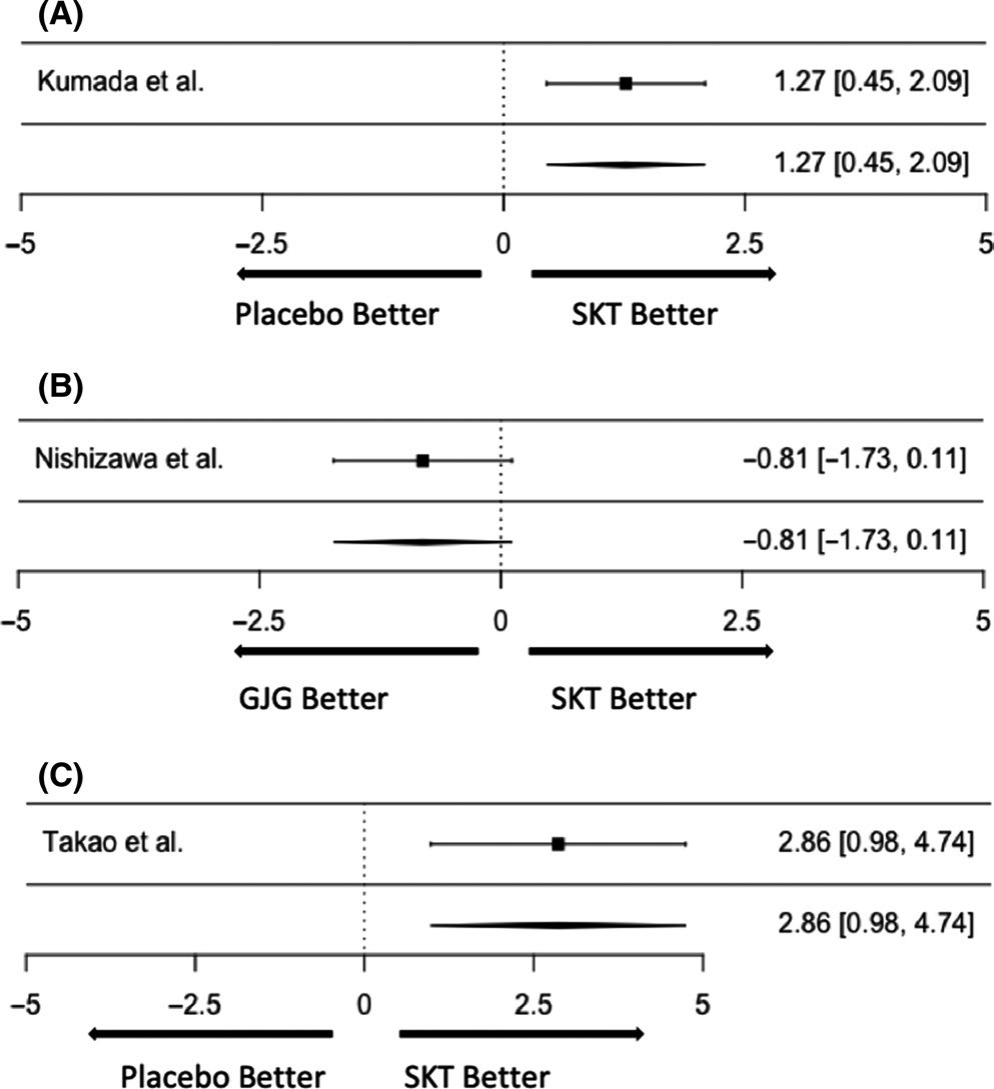
A, SKT vs Placebo. SKT, Shakuyaku‐kanzo‐to. B, SKT vs GJG. GJG, Goshajinkigan; SKT, Shakuyaku‐kanzo‐to. C, SKT vs Placebo. SKT, Shakuyaku‐kanzo‐to

Kumada et al^8^ reported that the improvement rate (“markedly improved” or “improved”) in terms of the frequency of muscle cramps was significantly superior with SKT than with placebo (Wilcoxon rank‐sum test, *P* = .011) in patients with liver cirrhosis. The OR for improvement comparing the SKT group to the placebo was 1.27 (95% confidence interval [CI]: 0.445‐2.086; Figure 2A). Nishizawa et al^9^ compared the effectiveness of SKT for painful muscle cramps associated with liver cirrhosis with that of GJG. Both are Japanese traditional herbal medicines and achieved improvements (“markedly improved” or “improved”) in the frequencies of muscle cramps. However, the improvement rate with GJG (60.5%) was significantly superior to that with SKT (40.5%; *P* < .05). The OR for improvement comparing the SKT group to the GJG group was −0.81 (95% CI, −1.734‐0.114; Figure 2B). Takao et al^10^ compared the effectiveness of SKT and eperisone hydrochloride in patients with lumbar spinal stenosis. They also sought to clarify the minimum effective dose of SKT. The improvement rate with SKT (87.5%) was higher than that with eperisone hydrochloride (28.57%), but statistical analysis was not performed. The OR for improvement comparing the SKT group with the eperisone hydrochloride group was 2.86 (95%CI, 0.980‐4.744; Figure 2C). The ORs of all three studies did not show statistical significance.

### 3.3 Adverse events

Kumada et al^8^ reported the incidence of adverse reactions was 14.3% with SKT and 4.9% with placebo. No significant difference was apparent between these two groups (Fisher's exact test: *P* = .173). Major adverse reactions included pseudoaldosteronism in SKT, but no severe adverse reactions were observed. Nishizawa et al^9^ reported the incidence of adverse reactions with SKT was 16.22% (pseudoaldosteronism in 1, myopathy in 1, and laboratory test abnormalities in 4), but with GJG was 0%. Takao et al^10^ reported one patient with dizziness in an SKT group. 

### 3.4 Quality assessment

The quality of the studies included in this review varied considerably. We evaluated the methodological quality of the three studies using the Cochrane risk‐of‐bias criteria and a modified version of the assessment checklist developed by Downs and Black (Tables 2 and 3). One study by Kumada et al^8^ was a randomized double‐blind placebo‐controlled parallel study, carrying a low risk of bias. The others lacked detailed descriptions of the methods, so we could not assess most domains.

**Table 2:** Summary of risk‐of‐bias assessment among the included studies

	Selection bias		Reporting bias	Performance bias	Detection bias	Attrition bias
	Random sequence generation	Allocation concealment				
Kumada et al^8^	Unclear	Low	Unclear	Low	Low	Low
Nishizawa et al^9^	Low	Unclear	Unclear	Unclear	Unclear	Unclear
Takao et al (2015)^10^	Unclear	Unclear	Unclear	High	High	Unclear

Note

According to the Cochrane Handbook for Systematic Review of Intervention.

**Table 3:** Summary of risk‐of‐bias assessment among the included studies

	Kumada et al^8^	Nishizawa et al^9^	Takao et al (2015)^10^
Reporting			
1. Is the hypothesis/aim/objective of the study clearly described?	Y	Y	Y
2. Are the main outcomes to be measured clearly described in the Introduction or Methods section?	Y	Y	Y
3. Are the characteristics of the patients included in the study clearly described?	Y	Y	Y
4. Are the interventions of interest clearly described?	Y	Y	Y
5. Are the distributions of principal confounders in each group of subjects to be compared clearly described?	P	N	N
6. Are the main findings of the study clearly described?	Y	Y	Y
7. Does the study provide estimates of the random variability in the data for the main outcomes?	Y	N	N
8. Have all important adverse events that may be a consequence of the intervention been reported?	Y	Y	Y
9. Have the characteristics of patients lost to follow‐up been described?	Y	N	N
10. Have actual probability values been reported?	Y	N	N
External validity			
11. Were the subjects asked to participate in the study representative of the entire population from which they were recruited?	Y	Unable	Unable
12. Were those subjects who were prepared to participate representative of the entire population from which they were recruited?	Y	Unable	Unable
13. Were the staff, places, and facilities where the patients were treated, representative of the treatment the majority of patients receive?	Y	Unable	Unable
Internal validity—bias			
14. Was an attempt made to blind study subjects to the intervention they have received?	Y	N	N
15. Was an attempt made to blind those measuring the main outcomes of the intervention?	N	N	N
16. If any of the results of the study were based on “data dredging”, was this made clear?	Y	Y	Y
17. In trials and cohort studies, do the analyses adjust for different lengths of follow‐up of patients, or in case‐control studies, is the time period between the intervention and outcome the same for cases and controls?	Y	Y	Y
18. Were the statistical tests used to assess the main outcomes appropriate?	Y	Unable	Unable
19. Was compliance with the intervention/s reliable?	Y	Unable	Unable
20. Were the main outcome measures used accurate (valid and reliable)?	Y	Y	Y
Internal validity—confounding (selection bias)			
21. Were the patients in different intervention groups recruited from the same population?	Y	Y	Y
22. Were study subjects in different intervention groups recruited over the same period of time?	Y	Y	Y
23. Were study subjects randomized to intervention groups?	Y	Y	Y
24. Was the randomized intervention assignment concealed from both patients and health care staff until recruitment was complete and irrevocable?	Y	Unable	N
25. Was there adequate adjustment for confounding in the analyses from which the main findings were drawn?	Unable	N	N
26. Were losses of patients to follow‐up taken into account?	Y	Unable	Unable

Note

According to a modified version of the assessment checklist developed by Downs and Black.

## 4 DISCUSSION

The present systematic review included only three clinical trials that investigated the efficacy of SKT against muscle cramps. These three studies had insufficient methodological quality, and we were thus unable to reach statistically valid conclusions. Although meta‐analysis could not be conducted because of the heterogeneity of the data and the small number of studies, SKT showed some clinical efficacy and safety in treating muscle cramps.

Shakuyaku‐kanzo‐to has significantly mitigated muscle symptoms in patients with a wide variety of underlying diseases. SKT has been found to show immediate efficacy against painful muscle cramps induced by liver cirrhosis, hemodialysis,^11^, ^12^ and diabetic neuropathy.^13^ SKT has been reported to inhibit acetylcholine‐induced and neurogenic contractions of the gastrointestinal tract and to decrease unfavorable smooth muscle contractions during upper and lower gastrointestinal endoscopy.^14^, ^15^, ^16^ SKT also inhibits oxytocin‐induced myometrial contractions of uterine tissue in pregnant women in a dose‐dependent manner.^17^, ^18^ In addition to ameliorating muscle symptoms, SKT has been found to improve extrapyramidal symptoms while exerting no significant effect on psychiatric symptoms.^19^ SKT has also been reported to ameliorate the myalgia and arthralgia induced by chemotherapy, using combination of paclitaxel and carboplatin in patients with non–small‐cell lung cancer^20^ as well as the oxaliplatin‐induced neurotoxicity in patients with metastatic colorectal cancer.^21^

In this systematic review, Nishizawa et al^9^ reported that GJG was superior to SKT in the treatment of painful muscle cramp and was safe in patients with cirrhosis. However, there was a paucity of methodological data evaluating bias. Poor inter‐rater reliability of the efficacy evaluations for both GJG and SKT was observed because of the lack of blinding. Pseudoaldosteronism is one of the most famous adverse events of SKT.^22^, ^23^ *R glycyrrhizae* (licorice) is the main component of SKT and has been reported to inhibit the conversion of cortisol to cortisone by 11β‐hydroxysteroid dehydrogenase and to cause pseudoaldosteronism.^22^, ^23^, ^24^ A study by Kumada et al^8^ reported that the frequency of hypokalemia of pseudoaldosteronism was lower when SKT was administered for 2 weeks. Prolonged intake of SKT for >30 days and age >60 years have been reported to increase the risk of hypokalemia with pseudoaldosteronism.^25^ SKT should thus be used as a rescue medicine or used within 14 days, because it can act quickly and the prolonged usage can potentially cause pseudoaldosteronism.

In conclusion, current evidence appears insufficient to allow adequate meta‐analysis of the effects of SKT, but SKT showed efficacy in the treatment of muscle cramps in patients with cirrhosis or lumbar spinal stenosis. Further randomized controlled trials with larger sample sizes are needed to assess the efficacy of SKT for muscle cramps.

## CONFLICT OF INTERESTS

The authors have stated explicitly that there are no conflicts of interest in connection with this article.

## AUTHORS CONTRIBUTIONS

KO designed the study and wrote the initial draft of the manuscript. KF contributed to analysis and interpretation of data, and assisted in the preparation of the manuscript. All other authors have contributed to data collection and interpretation, and critically reviewed the manuscript. All authors approved the final version of the manuscript and agree to be accountable for all aspects of the work in ensuring that questions related to the accuracy or integrity of any part of the work are appropriately investigated and resolved.

## ETHICS APPROVAL AND CONSENT TO PARTICIPATE

Not applicable.

## CONSENT TO PUBLISH

Not applicable.

## Data Availability

The datasets used and/or analyzed during the current study are available from the corresponding author on reasonable request.
